# The inhibition of Src kinase suppresses the production of matrix metalloproteinases in from synovial fibroblasts and inhibits MAPK and STATs pathways

**DOI:** 10.3906/sag-2008-274

**Published:** 2021-08-30

**Authors:** Demet YALÇIN KEHRİBAR, Metin ÖZGEN, Servet YOLBAŞ, Ahmet YILDIRIM, Neşe BAŞAK TÜRKMEN, Ebru ÖNALAN ETEM, Osman ÇİFTÇİ, İbrahim Hanifi ÖZERCAN, Süleyman Serdar KOCA

**Affiliations:** 1 Department of Internal Medicine, Faculty of Medicine, Ondokuz Mayıs University, Samsun Turkey; 2 Department of Rheumatology, Faculty of Medicine, Ondokuz Mayıs University, Samsun Turkey; 3 Department of Rheumatology, Faculty of Medicine, İnonu University, Malatya Turkey; 4 Department of Rheumatology, Elazığ Education and Research Hospital, Elazığ Turkey; 5 Department of Pharmaceutical Toxicology, Faculty of Pharmacy, İnönü University, Malatya Turkey; 6 Department of Medical Biology, Faculty of Medicine, Fırat University, Elazığ Turkey; 7 Department of Pharmacology, Faculty of Medicine, Pamukkale University, Denizli Turkey; 8 Department of Pathology, Faculty of Medicine, Fırat University, Elazığ Turkey; 9 Department of Rheumatology, Faculty of Medicine, Fırat University, Elazığ Turkey

**Keywords:** Rheumatoid arthritis, collagen induced arthritis, src kinase matrix metalloproteinase

## Abstract

**Background/aim:**

The purpose of this study was to investigate the antiarthritic potentials of the inhibition of Src kinase in vivo and in vitro settings.

**Materials and methods:**

Arthritis was induced by intradermal injection of chicken type II collagen combined with incomplete Freund’s adjuvant (collagen induced arthritis [CIA] model) in Wistar albino rats. One day after the onset of arthritis, dasatinib, a potent Src kinase inhibitor, (5 mg/kg/day) was given via oral gavage. Tissue Src, Fyn, MAPK and STAT mRNA expressions were determined by real-time polymerase chain reaction. On the other hand, fibroblast like synoviocytes (FLSs) were harvested patients with rheumatoid arthritis (RA) undergoing surgical knee joint replacement. FLSs were stimulated with cytokines and dasatinib was added in different concentrations. MMP –1, –3, and –13 levels in FLSs culture were determined by ELISA.

**Results:**

The tissue mRNA expressions of Src, Fyn, MAPK and STATs were increased in the arthritis CIA group compared to the control group. Their mRNA expressions in the CIA + dasatinib group were decreased and similar in the control group. In in vitro setting, MMP –1, –3, and –13 expressions from FLSs induced by IL-1β and TNF-α were increased, while dasatinib suppressed their productions from FLSs.

**Conclusion:**

The present study shows that the inhibition of Src kinase has antiarthritic potentials in both in vivo and in vitro settings. Src kinase inhibition may be candidate to further research in human RA.

## 1. Introduction

Rheumatoid arthritis (RA) is an inflammatory joint disorder [1–4]. Its pathogenesis is not fully known, yet. This disabling disease leads to chronic polyarthritis and joint deformities. Thus, it leads to a significant health and socioeconomic burden [1–4]. Although there is magnificent advances about the treatment of RA, disease modifying antirheumatic drugs (DMARDs) cannot prevent deformities and lead to clinical remission in all RA patients, as well as, several side effects of DMARDs can restrict the prescribe in some patients. Due to requirement for new therapeutic agents, biologic DMARDs such as tumor necrosis factor (TNF)-α inhibitors (TNFi), rituximab, abatacept, and tociluzumab are developed. Widening therapeutic options lead to reach radiologic and clinical remission in more patients. On the other hand, 30% of patients are achieved no responses, and some patients stop treatment due to adverse effects or secondary inadequate response. Thus, there is a requirement for the treatment of RA, yet. 

Src family kinases (SFKs) regulate cellular metabolism, survival, and proliferation. They act roles on the production of inflammatory cytokines/mediators [5,6]. SFKs, nonreceptor tyrosine kinases, include 9 structurally related molecules exhibiting similar functions. Src and Fyn belong to the SFKs. Both Src and Fyn exhibit ubiquitous expression, therefore they are involved in a variety of signal transduction pathways [7,8]. Thus, they play important roles in immune cell development, proliferation, adhesion, migration, chemotaxis, phagoscytosis, and survival. Moreover, Src and Fyn signaling result in the activation of signal transducers and activators of transcription (STAT) and mitogen-activated protein kinase (MAPK) which play pivotal roles in the pathogenesis of RA [1]. 

It has been reported that activated Src kinase is expressed by synovial cells and synovial macrophages in patients with RA [9]. Moreover, the depletion of Src kinase and SFKs has been shown to ameliorate the arthritis in rats [10]. Therefore, the purpose of this study was to investigate the antiarthritic potentials of the inhibition of Src kinase in vivo and in vitro settings.

## 2. Materials and methods

### 2.1. Animals

This study included 30 Wistar-albino female rats of 8–10 weeks old with weights varying between 220 and 260 g. Animals were housed in separate cages at a relative humidity rate of 70%–30% and a temperature rate of 24 ± 3 °C. A 12 h day: 12 h night cycle was followed. All rats were given ad libitum access to water. The sample size calculation is based on a previous study [11]. The study protocol was approved by the local animal ethic board (12.06.2013/78).

### 2.2. Experimental design

In this study, collagen induced arthritis (CIA) model was used to induce arthritis. The rats were divided into 3 groups: control group, arthritis (CIA) group, and dasatinib, which is a potent Src kinase inhibitor, treatment group. Each group consisted of 10 rats. Type 2 collagen (Sigma Aldrich Company, USA) was diluted with acetic acid (0.1 M concentration). Complete Freund’s adjuvant (Difco Laboratories, USA) was added to this solution. The solution was intradermally to the tail (100 µg) and each paws (total 200 µg to each animal). On the 7th day of the experiment, 100 µg booster doses were injected to each rat. Arthritis developing after collagen injection was scored separately for each rat. The severity of arthritis was scored according to the method previously described; score 0 was described as no erythema or edema, score 1 was described as mild erythema and edema in the foot or ankle, score 2 was described as mild erythema and edema in the paw, score 3 was described as moderate erythema and edema in the paw 3, score 4 was described as marked edema and anchylosis, and restricted movement in the paw [11,12]. After the onset of arthritis, dasatinib (5 mg/kg/day) was started to give via oral gavage, until the rats were sacrificed on day 29 [13].

### 2.3. Sample acquisition

After sacrificed, back paws were cut under the knee joint for histopathological evaluation and blood samples were taken for analyses. Joint samples were divided into two groups for the real-time polymerase chain reaction (PCR) analyses and histopathologic examinations [12,14]. The Hematoxylin-Eosin (H & E) stained lames were examined blindly by a pathologist for the histopathologic scoring of arthritis [12,14].

### 2.4. Real-time PCR analysis

According to the manufacturer’s total RNA from paws was extracted using Trizol reagent (Invitrogen, Carlsbad, CA, USA). cDNA was obtained by reverse transcription of total RNA samples with the cDNA Synthesis kit (Invitrogen, Carlsbad, CA, USA). Real time PCR reactions were performed in triplicate (heated to 50 °C for 2 min followed by 1 cycle of denaturation at 94 °C for 10 min, 40 cycles of 94 °C for 15 s, and 60 °C for 60 s). Standard curves were prepared for target genes and endogenous reference (HPRT) in each sample. The ABI Prism 7500 Fast Real Time PCR Instrument (Applied Biosystems, CA, USA) for real-time PCR analysis was used in this study with using Tag Man Master Mix (Applied Biosystems, CA, USA). All results are standardized according to GAPDH levels. The samples were quantified for MAPK, Fyn, Src, and STAT3 genes using the comparative Ct (ΔΔCt) protocol, according to the Assays–on-Demand User’s Manual (Applied Biosystems, CA, USA).

### 2.5. The inhibition of Src kinase in fibroblast-like synoviocytes cell culture 

Human FLS harvested from patients with RA were purchased from Sigma Aldrich (St. Louis, Missouri, USA). In this study, the FLS-RA were pre-exposed to pro-inflammatory cytokine those are IL-1β and TNF-α and initiated inflammation in chondrocytes [15]. The FLS-RA cultures were incubated for overnight with serum starved medium (0.5% FCS). Serum-starved the FLS-RA were pre -stimulated with 5 ng/mL IL-1β and 10 ng/mL TNF-α for 24 h Sigma Aldrich (St. Louis, Missouri, USA) before being treated with dasatinib (Bristol-Myers Squibb, Istanbul, Turkey). After overnight, various concentrations (1 to 20 μM) of dasatinib were administered to induce FLS - RA cell cultures and incubated for 24 h. To investigate effect of dasatinib in inflamed cells was studied MTS assays (Cambridge, UK) and ELISA assays. MMP –1, –3, and –13 levels in FLS-RA culture were determined by ELISA (Cambridge, UK).

### 2.6. Statistical analysis

The sample size of this study was eight per group and it was calculated based on an alpha error of 0.05 and a power of 80% and of 90% (GPower3.1). Data were analyzed using the Statistical Product and Service Solutions (SPSS, v: 22.0) software for windows. All data were presented as mean ± standard deviation. Kruskal–Wallis one-way analysis of variance and Mann–Whitney U test for dual-comparisons were used for statistical analysis. For categorical data analysis, spearman Chi-square test was used. Differences in arthritis severity scoring were analyzed by the Wilcoxon rank-sum test. P values less than 0.05 were considered significant.

## 3. Results

### 3.1. Clinical arthritis scoring

**Figure 1:**
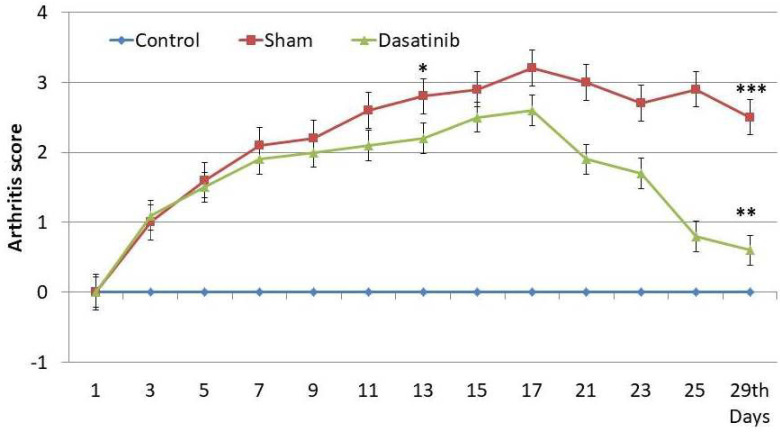
The scores of arthritis in the study groups *The mean arthritis scores were higher in the CIA group and CIA + dasatinib groups compared to the control group (p < 0.05). **The 29th day arthritis scores were decreased in the CIA + dasatinib group compared to the own 13th day score (p < 0.05). ***29th day arthritis score of CIA + dasatinib group was decreased compared to the 29th day score of CIA group (p < 0.05).

Joint swelling and erythema were observed at 12 to 13 days after the first injection of collagen in rats from the CIA group and CIA + dasatinib groups. The mean arthritis scores were higher in the CIA group and CIA + dasatinib groups compared to the control group (Figure 1). Dasatinib ameliorated arthritis. The mean 29th day arthritis scores were decreased in the CIA + dasatinib group compared to the own mean 14th day arthritis score (Wilcoxon Rank p < 0.001). Moreover, the 29th day arthritis score of CIA + dasatinib group was decreased compared to the mean 29th day arthritis score of CIA group (p < 0.001) (Figure 1). 

### 3.2. Pathological examinations

**Figure 2:**
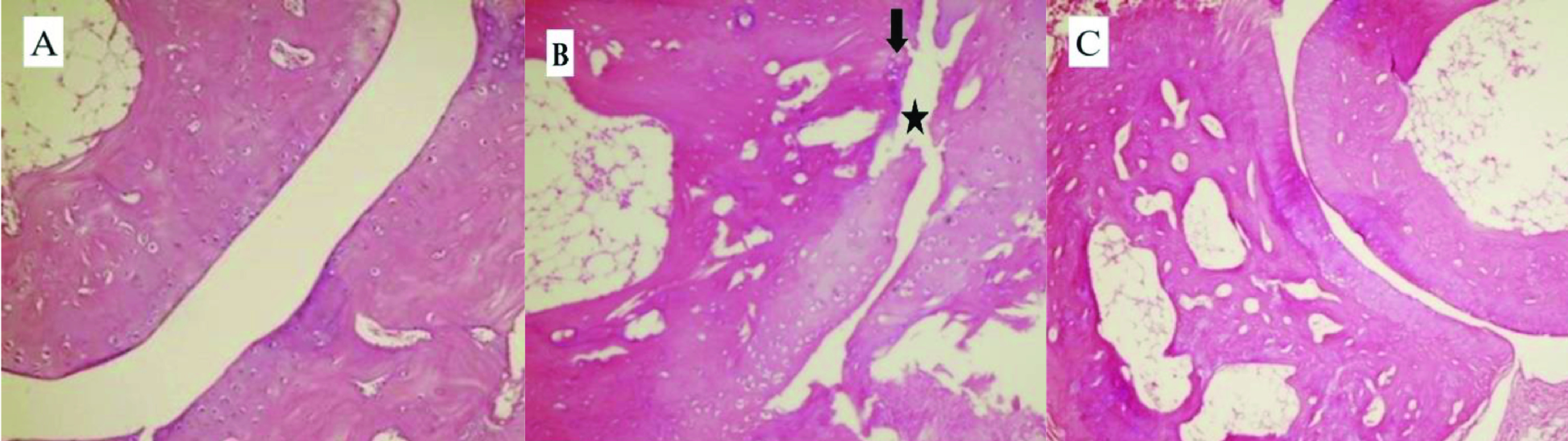
. Histopathological sections of joints in study groups (H&E X400). Histopathological appearance of the joint was normal in the control group (A). Obvious perisynovial inflammation (arrow) and destruction of cartilage-bone (star) were observed in the arthritis CIA group (B). Perisynovial inflammation and synovial hyperplasia were decreased in the CIA + dasatinib group (C).

The extensive perisynovial inflammation and marked cartilage - bone destruction in the CIA group rats were demonstrated by histopathological analysis (Figure 2A and Figure 2B). On the other hand, dasatinib ameliorated the perisynovial inflammation and cartilage-bone destruction in the paws (Figure 2C). 

### 3.3. mRNA expressions from joint tissues

The tissue mRNA expressions of Src (Figure 3A), Fyn (Figure 3B), MAPK (Figure 3C) and STAT3 (Figure 3D) (for all, p < 0.001) were increased in the CIA group compared to the control group. However, dasatinib treatment suppressed the tissue mRNA expressions of Src, Fyn, MAPK (p = 0.009), and STAT3 (for all, p < 0.001). Their mRNA expressions in CIA + dasatinib group were similar in the control group (p > 0.05). 

### 3.4. The productions of MMPs from FLS-RA

In in vitro setting, IL-1β, and TNF-α induced FLS-RA. The applications of IL-1β and TNF - α on FLS-RA culture increased the production of MMP –1, –3, and –13 (p < 0.05). On the other hand, dasatinib suppressed the productions of MMP–1 ((Figure 4A), MMP–3 (Figure 4B), and MMP–13 (Figure 4C) from FLS-RA induced by IL-1β and TNF-α (p < 0.05).

## 4. Discussion

In the present study, the effects of Src and Fyn kinase inhibition by dasatinib were researched on CIA and cell culture of FLS. The mRNA expressions of Src and Fyn kinases were increased in the rats with clinically and histopathologically evident arthritis. When the rats with arthritis were treated with dasatinib, clinical and histopathological findings of arthritis are ameliorated, as well as, the mRNA expressions of Src and Fyn kinases were decreased, in our study. Moreover, in in vitro setting, the induction of FLS by IL-1 and TNF-α increased the production of MMPs. When dasatinib was applied to IL-1 and TNF-α induced FLS, the levels of MMPs were decreased, in our study.

Src-family kinases affect various cellular signaling pathways, those regulate the motility, survival, proliferation, adhesion, and invasion of cells [6–8]. It has been reported that activated Src is expressed by synovial cells and synovial macrophages in patients with RA [9]. Moreover, the depletion of Src and SFKs has been shown to ameliorate the arthritis in rats [10]. In the present study, it was shown that Src and Fyn mRNA expressions are increased in the rats with arthritis, and dasatinib a SFK inhibitor ameliorates arthritis and depletes the expressions of Src and Fyn in CIA model. Moreover, dasatinib decreases the production of MMP from FLS in in vitro setting. These results may suggest that SFKs have prominent roles on the pathogenesis of RA.

The p38 MAPK pathway in RA plays pivotal roles [16]. Activation of p38 MAPK triggers the production of several pro-inflammatory cytokines such as TNF, IL-1 and IL-6 in synovium and the inhibition of p38 MAPK with a synthetic inhibitor has decreased inflammation [17]. Moreover, recent accumulated evidences demonstrate that the p38 MAPK pathway has a central role in emergence of cartilage and bone destruction in RA through synthesis of MMPs, formation of osteoclasts, and angiogenesis [16,17]. On the other hand, several reports show that SFKs are associated with the p38 MAPK pathway [18–20]. Summy et al. [18] have showed the p38 MAPK activation is regulated by Src kinase. Another study has demonstrated that the inhibition of Src activation with a SFK inhibitor reduced p38 MAPK activation [20]. And they reported that Src kinase is essential for the activation of p38 MAPK in spinal microglia cell culture [20]. In the present study, it has been observed that MAPK mRNA expression is increased in CIA, while dasatinib ameliorates arthritis and depletes the mRNA expression of MAPK in this experimental arthritis model. These results suggest that the relationship between SFK and MAPK has a role in inflammatory arthritis.

STATs are a family of cytoplasmic transcription factors, they are activated when critical tyrosine residues become phosphorylated by tyrosine kinases. When the tyrosine residues of STAT proteins become phosphorylated, STATs translocate to the nucleus. And thus, they bind to cognate sequences contained within gene promoters and regulate gene expression [21]. Numerous pro-inflammatory cytokines are increased in RA and they activate STAT signaling pathway. Activation of STAT is one of the leading actors in the pathogenesis of RA [22]. In RA, the increased activity of STAT3 in inflammatory cells and synovial fibroblasts has been documented and the STAT3 is associated with the development of the disease [22]. Oike et al. [23] have shown that STAT3 inhibitors ameliorate arthritis in CIA model. On the other hand, Schreiner et al. [24] have demonstrated that Src kinases activate STAT3 in in vitro setting. In our study, dasatinib decreased the mRNA expressions of Src kinase and STAT3 from joint tissues. This result supports that the effect of Src kinase on inflammatory arthritis may also be related with their actions on STATs. 

Kovacs et al. [25] have documented that knockout mice for Src kinases are protected from inflammatory cell infiltrations in an experimental arthritis model. It is due to defective productions of cytokines and chemokines by inflammatory cells infiltrating inflame joints [25]. Dasatinib, a Src kinase inhibitor, is shown to inhibit the production of TNF-α, IL-6, and IL-8 from macrophages, monocytes and RA-synovial fibroblasts as well as dasatinib affects the activations of these cells [26]. In our study, dasatinib decreased the productions of MMPs from RA-FLS. Kramer et al. [27] have documented that dasatinib reduces the production of MMM-9 from 3 different cell lines originating from patient with squamous cell carcinoma. In RA, pro-inflammatory cytokines affect FLSs and macrophages to release MMPs those are responsible to joint destructions [28]. In our study, it has been observed that dasatinib decreased the productions of MMPs from RA-FLSs.

There are some limitations in the present study. We assessed the expressions of Src, Fyn, MAPK, and STAT mRNA. But, they do not indicate their protein levels and activities. Moreover, it would have been better if erythrocyte sedimentation rate, C-reactive protein level, and complete blood count were also evaluated.

Dasatinib is a second generation tyrosine-kinase inhibitor to treat chronic myeloid leukemia. The present study shows that dasatinib has antiarthritic potentials in both in vivo and in vitro settings. It may be candidate to further research in human RA.

## Author contributions

All authors make substantial contributions to conception and design, and/or acquisition of data, and/or analysis and interpretation of data.

## Funding

This study supported by Fırat Üniversitesi Bilimsel Araştırma Projeleri (TF.13.53).
